# Long non-coding RNA pairs to assist in diagnosing sepsis

**DOI:** 10.1186/s12864-021-07576-4

**Published:** 2021-04-16

**Authors:** Xubin Zheng, Kwong-Sak Leung, Man-Hon Wong, Lixin Cheng

**Affiliations:** 1 Shenzhen People’s Hospital, First Affiliated Hospital of Southern University of Science and Technology, Second Clinical Medicine College of Jinan University, Shenzhen, 518020 China; 2 Department of Computer Science and Engineering, The Chinese University of Hong Kong, Shatin, New Territories Hong Kong

**Keywords:** Sepsis, Diagnostics, Signature, Long non-coding RNA, Relative expression

## Abstract

**Background:**

Sepsis is the major cause of death in Intensive Care Unit (ICU) globally. Molecular detection enables rapid diagnosis that allows early intervention to minimize the death rate. Recent studies showed that long non-coding RNAs (lncRNAs) regulate proinflammatory genes and are related to the dysfunction of organs in sepsis. Identifying lncRNA signature with absolute abundance is challenging because of the technical variation and the systematic experimental bias.

**Results:**

Cohorts (*n* = 768) containing whole blood lncRNA profiling of sepsis patients in the Gene Expression Omnibus (GEO) database were included. We proposed a novel diagnostic strategy that made use of the relative expressions of lncRNA pairs, which are reversed between sepsis patients and normal controls (eg. lncRNA_*i*_ > lncRNA_*j*_ in sepsis patients and lncRNA_*i*_ < lncRNA_*j*_ in normal controls), to identify 14 lncRNA pairs as a sepsis diagnostic signature. The signature was then applied to independent cohorts (*n* = 644) to evaluate its predictive performance across different ages and normalization methods. Comparing to common machine learning models and existing signatures, SepSigLnc consistently attains better performance on the validation cohorts from the same age group (AUC = 0.990 & 0.995 in two cohorts) and across different groups (AUC = 0.878 on average), as well as cohorts processed by an alternative normalization method (AUC = 0.953 on average). Functional analysis demonstrates that the lncRNA pairs in SepsigLnc are functionally similar and tend to implicate in the same biological processes including cell fate commitment and cellular response to steroid hormone stimulus.

**Conclusion:**

Our study identified 14 lncRNA pairs as signature that can facilitate the diagnosis of septic patients at an intervenable point when clinical manifestations are not dramatic. Also, the computational procedure can be generalized to a standard procedure for discovering diagnostic molecule signatures.

**Supplementary Information:**

The online version contains supplementary material available at 10.1186/s12864-021-07576-4.

## Background

Sepsis is a severe disease that threatens patients’ life and is the main cause of death in Intensive Care Unit (ICU). Based on the report from Centers for Disease Control and Prevention, over 1.7 million people get sepsis each year, up to 270,000 Americans are killed by the disease per year, and one in three patients who die in a hospital have sepsis [1]. It is occasioned by the over response of the immune system to the infection. The chemicals released by the immune system diffuse throughout all the body and lead to inflammation. Septic shock is a subtype of sepsis when the blood circulation and cellular metabolism of the patients become deadly abnormal [2]. The diagnosis of sepsis in clinic bases on the symptoms and a series of medical tests involving blood, urine, wound secretion, and mucus secretion tests. Taking these typical tests may result in delays in diagnosis and intervention. Besides, it is challenging to distinguish sepsis from non-infectious inflammation based on existing tests [3].

Molecular detection provides a rapid way for early diagnosis and initial evaluation of sepsis. Moreover, it can determine whether a patient with systemic inflammation has an underlying infection by identifying biomarkers or signatures for sepsis. Procalcitonin (PCT) is widely studied as a “standard of care” component for sepsis and has been applied in blood tests for quick diagnosis [4]. However, some studies on PCT in intra-abdominal infections led to contradictory results and restricted the application of PCT as a diagnostic biomarker [5–7]. Protein-coding genes are also investigated as diagnostic signatures for sepsis with high accuracy according to a series of in silico experiments [8]. sNIP [9] and SeptiCyte Lab [10] are the two most effective genetic signatures for sepsis diagnosis proposed recently. SeptiCyte Lab is based on the sum of two ratio classifiers, each of which consists of a ratio of two genes (PLAC8/PLA2G7 or LAMP1/CEACAM4) and performed AUC of 0.92 in average in three validation cohorts while PCT only attained AUC of 0.81. It was regarded as the benchmark of sepsis diagnosis [11]. Scicluna et al. identified three genes and developed the sNIP score [(NLRP1 - IDNK)/PLAC8] from a discovery cohort including 60 abdominal sepsis patients and 42 controls. sNIP achieved AUC of 0.97 in the discovery cohort, which outperformed SeptiCyte (AUC = 0.89).

Long non-coding RNA (lncRNA) is a category of RNAs longer than 200 base pairs in length with little potential of encoding proteins [12]. lncRNAs are involved in the mediation of transcriptional and post-transcriptional regulation, which is a canonical epigenetic mechanism of cell dysfunction linked to a variety of immune-related diseases [13–15]. Ho et al. reviewed the regulatory non-coding RNAs in sepsis including lncRNA MIR210HG, linc-ATP13A4–8, linc-KIAA1737–2, AL132709.5, CTC-459I6.1 and IL7R [16]. Reddy et al. identified lncRNA E33 to regulate expression of inflammation related gene in macrophage and the response to the inflammatory signals through diabetic mice model [17]. Chen et al. investigated MALAT1 in rat sepsis model and found it regulates sepsis-induced cardiac inflammation [18]. Q. Huang et al. [19] and S. Huang et al. [20] showed that NEAT1 is overexpressed in sepsis patients and the expression level is correlated to severity of sepsis. Furthermore, ZFAS1 was detected to be downregulated in sepsis patients comparing to normal controls and achieved an Area Under the Receiver Operating Characteristic (AUROC) of 0.814 in sepsis diagnosis [21].

At present, machine learning methods are widely used for the biological modeling and biomarker detection. For instance, Wang et al. reviewed the machine learning tools in CRISPR gRNA design [22] and proposed GNL-Scorer to predict the CRISPR on-target activity with well cross-species generalization using Gradient Boosted Regression Tree and Bayesian Ridge regression [23]. B. Elkarami et al. proposed a cost-sensitive classifier and ensembling based on random forest method for handling the imbalanced quantity of miRNAs versus other non-coding RNA [24]. Liu et al. applied decision tree to identify gut microbial biomarkers as potential therapeutic target for atherosclerosis patients [25]. Liu et al. identified a 28-lncRNA signature for sepsis diagnosis using least absolute shrinkage and selection operator (LASSO) [26].

To the best of our knowledge, all the sepsis related transcriptome studies are based on the absolute expression abundance of the detected transcripts, e.g., mRNAs or lncRNAs. Nevertheless, using the absolute abundance for diagnosis is not reliable and even biased due to the systematic experimental bias and technical variation. Moreover, the investigation of biomarkers using absolute expression values may suffer from batch effect and the effect of pre-processing methods. Here, we proposed a signature for sepsis diagnosis using the relative expressions of lncRNA pairs within sample. We firstly performed the intra-sample comparison to obtain the relative expressions of all possible lncRNA pairs and conducted cross-sample analysis to screen out lncRNA pairs that significantly altered in direction between sepsis and control samples. Then, the identified lncRNA signature (lncRNA pairs) was evaluated in eight sepsis expression cohorts and compared with a series of machine learning models and benchmark signatures. Finally, these lncRNA pairs were investigated for their biological functions using enrichment and semantic analysis.

## Material and methods

### Expression datasets

We collected nine sepsis expression datasets with normal controls from the Gene Expression Omnibus (GEO) database. Only the platform Affymetrix Human Genome U133 Plus 2.0 Array (AffyU133p2) was considered, because it is the most comprehensive array platform with the largest transcript coverage, which is able to reannotate as many lncRNAs as possible from the arrays originally designed for coding genes. Detailed description for each dataset was shown in Table 1. For fair comparison, all the raw data were normalized by the Robust Multi-array Average (RMA) method [35] and MicroArray Suite 5.0 (MAS5.0) [36], although the normalization step is not necessary for our proposed algorithm [37].

**Table 1:** Datasets included in the analysis

Dataset accession	Cohort description	Sample type	Number of Septic subjects	Number of Normal subjects	Total subjects	Reference
**Discovery cohort**						
GSE95233	Adults	Whole blood	102	22	124	Ref. [27]
**Validation Cohorts**						
GSE57065	Adults	Whole blood	82	25	107	Ref. [28]
GSE28750	Adults	Whole blood	10	20	30	Ref. [29]
GSE8121	Children	Whole blood	60	15	75	Ref. [30]
GSE9692	Children	Whole blood	30	15	45	Ref. [31]
GSE13904	Children	Whole blood	52	18	70	Ref. [32]
GSE26378	Children	Whole blood	82	21	103	Ref. [33]
GSE4607	Children	Whole blood	69	15	84	Ref. [34]
GSE26440	Children	Whole blood	98	32	130	Ref. [33]

### Reannotation of lncRNAs

Based on the annotation files of the RefSeq database (release 79), the NetAffx file (release 36, 7/12/16), and the GENCODE project (release 25), the probes in the AffyU133p2 array platform were reannotated to achieve the expression profiles of lncRNAs [12]. Expression data of 3746 lncRNAs summarized by 4602 probe sets were obtained according to the selection criteria, (1) probe sets with RefSeq IDs labeled “NR_” and annotated with “long non-coding RNA” in the RefSeq; (2) probe sets with Ensembl gene IDs annotated as “long non-coding RNA” in GENCODE. Probe sets meet any of the two criteria were recruited as lncRNAs.

### iPAGE

**Fig. 1:**
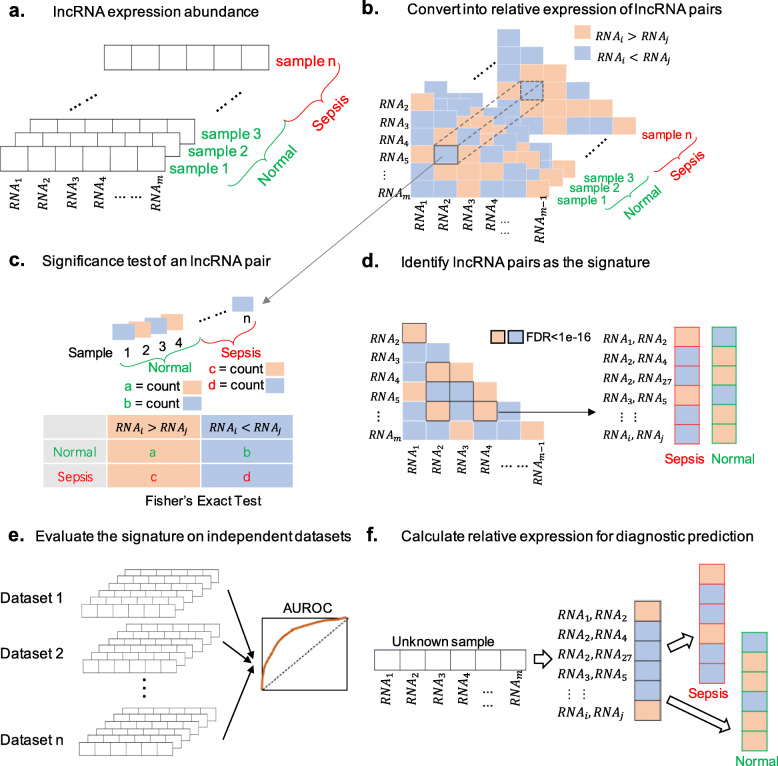
The workflow of individualized pair analysis of gene expression (iPAGE). **a** The long non-coding RNA (lncRNA) expression data including both sepsis and normal samples. **b** Relative expression between every possible lncRNA pairs were calculated in each sample. **c** Statistical analysis across all samples in the discovery cohort. **d** Top lncRNA pairs with significant differentiation ability were identified as the signature. **e** Validation using independent datasets. **f** Diagnostic application of the proposed signature

An algorithm, individualized Pair Analysis of Gene Expression (iPAGE), was used to screen the differential discoveries. The absolute expression abundance of genes is frequently varied by plenty of technical variations including experimental designs, sample handling, RNA amounts and extraction procedures, library preparation steps, as well as normalization methods and batch effects. As evidenced by the previous studies [38, 39], the relative expression between genes within a sample is reliable and much more powerful in detecting biological signals. So, we took advantage of the expression levels between every possible pair of lncRNAs to retrieve disease-related lncRNA pairs. The reverse pairs are defined as the lncRNA pairs with the same relative expression order (lncRNA_*i*_ > lncRNA_*j*_) in normal cases while the opposite order (lncRNA_*i*_ < lncRNA_*j*_) in sepsis patients. After that, we identified the top reverse lncRNA pairs and used them as a signature for the diagnostic prediction of sepsis. The workflow of iPAGE is described in Fig. 1.

In our iPAGE algorithm, exhaustive comparisons were carried out between every lncRNAs based on their absolute abundance. As shown in Fig. 1a, the lncRNAs detected by a sample are represented by a vector ${G}^{(k)}=\left({RNA}_1^{(k)},{RNA}_2^{(k)},\dots, {RNA}_m^{(k)}\right)$, where ${RNA}_1^{(k)},{RNA}_2^{(k)},\dots, {RNA}_m^{(k)}$ are the absolute abundance of lncRNAs and the superscript (*k*) represents the k-th sample in a given dataset. The relative expression of a lncRNA pair $\left({RNA}_i^{(k)},{RNA}_j^{(k)}\right)$ is defined as

1 $$ {r}^{(k)}=I\left({RNA}_i^{(k)}-{RNA}_j^{(k)}\right) $$

$$ \mathrm{where}\ \mathrm{I}(x)=\left\{\begin{array}{c}1, if\ x\ge 0\\ {}-1, if\ x<0\end{array}\right. $$ is an indicator revealing whether *x* is greater or less than zero. If ${RNA}_i^{(k)}$ is greater than or equal to ${RNA}_j^{(k)}$, the relative expression of the lncRNA pair $\left({RNA}_i^{(k)},{RNA}_j^{(k)}\right)$ will be 1. Otherwise, the relative expression will be − 1. To convert the absolute expression abundance into relative expression within each sample, subtraction was performed between every two lncRNAs (Fig. 1b), which was ${RNA}_i^{(k)}-{RNA}_j^{(k)},\forall i,j\in \left\{1,\dots, \mathrm{m}\right\},\mathrm{i}\ne \mathrm{j}$. *R*^(*k*)^ is a vector constituted by the relative expressions of all lncRNA pairs within the k-th sample (Eq. 2).

2 $$ {\displaystyle \begin{array}{l}{R}^{(k)}=\left({r}_{12}^{(k)},{r}_{13}^{(k)},\dots, {r}_{1m}^{(k)},{r}_{23}^{(k)},{r}_{24}^{(k)},\dots, {r}_{2m}^{(k)},\dots, {r}_{\left(m-1\right)m}^{(k)}\right)\\ {}=\Big(I\left({RNA}_1^{(k)}-{RNA}_2^{(k)}\right),I\left({RNA}_1^{(k)}-{RNA}_3^{(k)}\right),\dots, I\left({RNA}_1^{(k)}-{RNA}_m^{(k)}\right),\\ {}\begin{array}{l}\dots, I\left({RNA}_2^{(k)}-{RNA}_3^{(k)}\right),I\left({RNA}_2^{(k)}-{RNA}_4^{(k)}\right),\dots, I\left({RNA}_2^{(k)}-{RNA}_m^{(k)}\right),\\ {}\dots, I\left({RNA}_{m-1}^{(k)}-{RNA}_m^{(k)}\right)\Big)\end{array}\end{array}} $$

*R*^(*k*)^ is in ${C}_m^2$ dimensions, as there are totally ${C}_m^2=\frac{m!}{2!\left(m-2\right)!}$ lncRNA pairs.

The relative expression *R*^(*k*)^ of lncRNAs together with label *Y*^(*k*)^ forms a group of training data S = {(*R*^(1)^, *Y*^(1)^), (*R*^(2)^, *Y*^(2)^), …, (*R*^(*n*)^, *Y*^(*n*)^)}, where *Y*^(*k*)^ equals to 0 for a normal sample or 1 for a sepsis sample. The relative expression of lncRNAs pairs within sample is stable, even though the absolute expression value may bias among samples ubiquitously. After the relative expression within sample extracted, the cross-sample analysis was conducted following the steps below.

To screen out the intra-sample signature for sepsis, cross-sample analysis was performed between sepsis and normal samples (Fig. 1c). The lncRNA pairs with a significant difference in their relative expression ${r}_{ij}^{(k)}$ between sepsis and normal samples were extracted. Two situations, ${r}_{ij}^{(k)}=1$ (${RNA}_i^{(k)}>{RNA}_j^{(k)}$) and ${r}_{ij}^{(k)}=-1$ (${RNA}_i^{(k)}<{RNA}_j^{(k)}$), were taken into consideration. For each lncRNA pairs in the normal group, the number of *RNA*_*i*_ > *RNA*_*j*_ across n samples was calculated as

3 $$ \mathrm{a}=\frac{1}{2}{\sum}_{k=1}^n\left({r}_{ij}^{(k)}+1\right)\times \left(1-{Y}^{(k)}\right), $$

and the number of *RNA*_*i*_ < *RNA*_*j*_ is

4 $$ \mathrm{b}=\frac{1}{2}{\sum}_{k=1}^n\left(1-{r}_{ij}^{(k)}\right)\times \left(1-{Y}^{(k)}\right). $$

For the sepsis group, the number of *RNA*_*i*_ > *RNA*_*j*_ is

5 $$ \mathrm{c}=\frac{1}{2}{\sum}_{k=1}^n\left({r}_{ij}^{(k)}+1\right)\times {Y}^{(n)}, $$

while *RNA*_*i*_ < *RNA*_*j*_ is

6 $$ \mathrm{d}=\frac{1}{2}{\sum}_{k=1}^n\left(1-{r}_{ij}^{(k)}\right)\times {Y}^{(n)}. $$

Hence, the contingency table was obtained as follows,

**Table:** 

	${RNA}_i^{(k)}>{RNA}_j^{(k)}$	${RNA}_i^{(k)}<{RNA}_j^{(k)}$
Normal	$\mathrm{a}=\frac{1}{2}\sum \limits_{k=1}^n\left({r}_{ij}^{(k)}+1\right)\times \left(1-{Y}^{(k)}\right)$	$\mathrm{b}=\frac{1}{2}\sum \limits_{k=1}^n\left(1-{r}_{ij}^{(k)}\right)\times \left(1-{Y}^{(k)}\right)$
Sepsis	$\mathrm{c}=\frac{1}{2}\sum \limits_{k=1}^n\left({r}_{ij}^{(k)}+1\right)\times {Y}^{(n)}$	$\mathrm{d}=\frac{1}{2}\sum \limits_{k=1}^n\left(1-{r}_{ij}^{(k)}\right)\times {Y}^{(n)}$

After that, Fisher’s Exact Test was utilized to measure the ability of differentiating sepsis ones from the control samples for each lncRNA pair. The *p* value was calculated by

7 $$ \mathrm{p}=\frac{\left(\begin{array}{c}a+b\\ {}a\end{array}\right)\left(\begin{array}{c}c+d\\ {}c\end{array}\right)}{\left(\begin{array}{c}n\\ {}a+c\end{array}\right)}=\frac{\left(a+b\right)!\left(c+d\right)!\left(a+c\right)!\left(b+d\right)!}{a!b!c!d!n!} $$

, where n = a + b + c + d.

Bonferroni correction was then applied for multiple comparison correction. Base on the adjusted *p* values, the significantly altered lncRNA pairs (SALPs) were screened out between normal samples and septic samples as the signature (Fig. 1d). In this study, the SALPs *r*_*ij*_ was selected with ${p}_{r_{ij}}$ smaller than 1 × 10^−16^.

### Classification and evaluation

lncRNA pairs selected in the previous step may serve as a diagnostic signature. A classifier was built based on the relative expressions of lncRNA pairs, which were represented by *r*_1_, *r*_2_, …, *r*_*l*_ for simplification. r is assigned 1 when a pair *RNA*_*i*_ > *RNA*_*j*_ indicates sepsis and r is assigned −1 otherwise. A risk score ρ indicating the sepsis possibility was calculated by taking the sum of all the differential lncRNA pairs, which is $\uprho =\frac{1}{2}{\sum}_{q=1}^l\left({r}_q+1\right).$ The classifier was evaluated by Area Under the Receiver Operating Characteristic (AUROC) on eight independent validation cohorts (Fig. 1e) and then can be applied for diagnosis prediction (Fig. 1f). All the above experiments were conducted using Python 2.7.

### Functional analysis

The interactions between lncRNAs and proteins were obtained from the RNAinter database [40]. Proteins interacted with a specific lncRNA were assumed to be involved in the similar biological functions with the lncRNA. We used hypergeometric test to evaluate the statistical significance of functional enrichment, mining functional terms or pathways that a set of proteins are overrepresented [41–43]. The functional similarity between two sets of genes or proteins was measured by semantic similarity using Wang’s algorithm [44]. These calculations were carried out using clusterProfiler [45] and GOSemSim [46] in R environment.

## Results

### Data curation

We performed a systematic search for microarray datasets that collected whole blood from sepsis samples and identified three adult datasets and six children’s datasets (Table 1). The dataset GSE95233 with most sepsis samples was selected as discovery cohort. The other two adult datasets served as independent validation sets for the same group and six children’s dataset were utilized for divergent group validation. After Robust Multiarray Averaging (RMA) [35] normalization and reannotation, 3745 lncRNAs were obtained for individualized pair analysis of gene expression (iPAGE) to identify the significantly altered lncRNA pairs (SALPs). Moreover, the eight validation datasets normalized by MicroArray Suite 5.0 (MAS5.0) [36] were applied to evaluate the model performance across distinct normalization methods.

### Intra-sample signature discovery

**Fig. 2:**
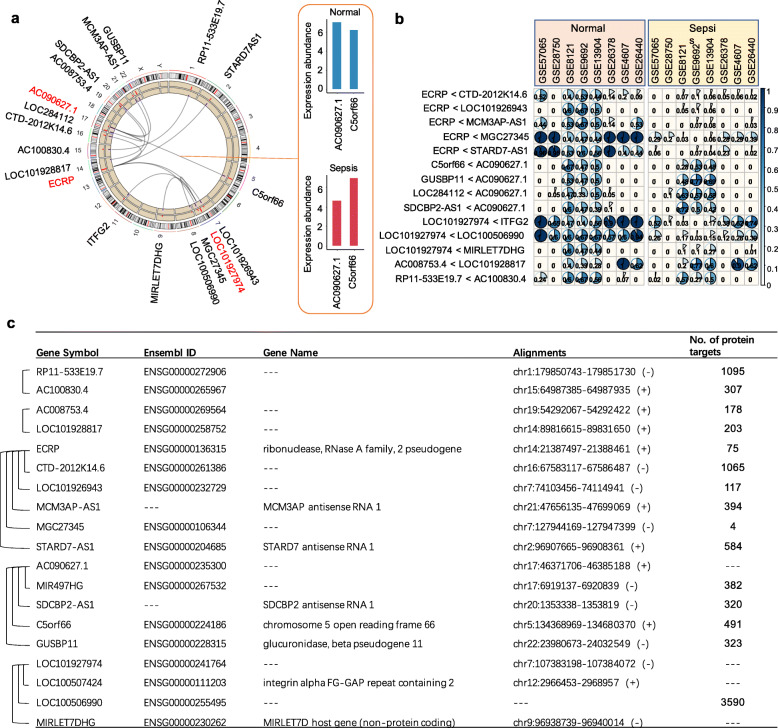
Statistics and characteristics of 14 lncRNA pairs in SepSigLnc. **a** A circular plot showing the location of the identified lncRNA pairs in chromosome. Nodes represent lncRNAs and edges represent the lncRNA pairs between sepsis and normal states. Red and blue bars represent the mean absolute abundance in discovery cohort. **b** A grid of pie charts showing the percentage of the lncRNA pairs’ relative expression between sepsis and normal samples in the eight validation cohorts. Each pie represents the percentage of samples in a cohort whose lncRNA pair follow the relative expression (equal to − 1) shown on the left. **c** The genetic information of the lncRNAs in SepSigLnc

A total of 7,010,640 intra-sample lncRNA pairs were acquired from 3745 lncRNAs using exhaustive comparison between every lncRNA. Among these pairs, 14 lncRNA pairs are the most significantly different between the septic patients and control samples after performing cross-sample analysis (*P* value < 1 × 10^−16^, Fisher’s Exact test). The 14 lncRNA pairs are named SepSigLnc and can be used as a transcriptional signature for sepsis diagnosis (listed in Fig. 2b). In the discovery cohort, the lncRNAs on the left are smaller than the ones on the right column in expression level among all the normal samples, whereas the relative expression reverse in the sepsis samples, i.e., from *RNA*_*i*_ < *RNA*_*j*_ to *RNA*_*i*_ > *RNA*_*j*_. Three lncRNAs are involved in multiple pairs among the 14 pairs, resulting in 19 individual lncRNAs. Specifically, *ECRP*, *AC090627.1*, and *LOC101927974* are implicated in five, four, and three pairs, respectively.

A circos plot shows the location of SepSigLnc on chromosomes with each line linking a pair of lncRNAs (Fig. 2a). Mean expression levels of lncRNAs in normal and sepsis samples among discovery cohort are displayed in blue and red bars, respectively. The expression levels of lncRNAs in a pair reverse between sepsis and control samples. For instance, *C5orf66* has higher expression level than *AC090627.1* among the sepsis patients, whereas it expresses lower than *AC090627.1* among the normal samples. For the eight validation cohorts, the identified 14 lncRNA pairs illustrate distinct expression patterns between normal and sepsis samples (Fig. 2b). Specifically, the proportion of samples with relative expression equal to − 1 among normal samples is no less than that among sepsis patients in each cohort. In another aspect, normal individuals have fewer reverse lncRNA pairs than sepsis ones (Supplementary Figure S2 in Additional file). Figure 2c provides the detailed information of the 19 lncRNAs in SepSigLnc.

### Model comparison

**Fig. 3:**
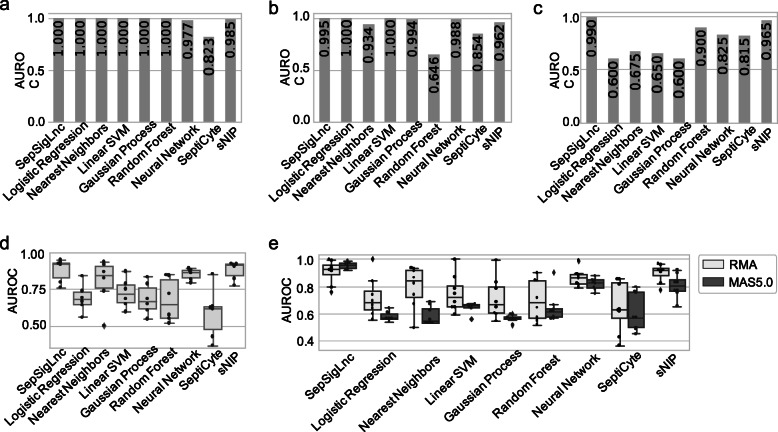
AUROC of SepSigLnc, machine learning methods, and benchmarks in discovery cohort and validation data sets. **a** AUROC of SepSigLnc, machine learning methods, and benchmarks on discovery cohort GSE95233. **b** Performance on adult validation cohort GSE57065 measured by AUROC. **c** Performance on adult validation cohort GSE28750 measured by AUROC. **d** Boxplots of performance in six children’s validation cohorts measured by AUROC. **e** Boxplots of performance measured by AUPRC on totally eight validation cohorts normalized with RMA and MAS5.0

For comparison, we also trained machine learning methods on the discovery cohort including logistic regression, nearest neighbors classifier, linear support vector machine (SVM), gaussian process classification (GPC), random forest, and neural network. As the workflow presented in Supplementary Figure S1 (see Additional file), the top 19 significantly differential lncRNAs, the same number of lncRNAs as SepSigLnc, were extracted by independent student’s T-test as features for machine learning models. After tuning with 10-fold cross validation and grid search, the optimal number of estimators for random forest was 10, maximum depth was 2. As for neural network, two hidden layers with 20 neurons for each layer had the best performance through grid search. Also, we compared SepSigLnc with existing genetic signatures SeptiCyte [10] and sNIP [9]. The AUROCs for all the methods on the discovery cohort are shown in Fig. 3a.

### Validation by adult data sets

GSE57065 and GSE28750, the same age group as the training cohort, were employed as independent validation cohorts to test the performance of SepSigLnc on adults. SepSigLnc achieved the AUROC of 0.995 on GSE57065 and AUROC of 0.990 on GSE28750 (Fig. 3b and c). We compared the performance of SepSigLnc with other machine learning methods using the same number of lncRNAs. Logistic regression and linear SVM had the highest AUROC of 1 on GSE57065, while the SepSigLnc achieved a comparable result of 0.995. Nevertheless, SeptiCyte and sNIP performed not as well as SepSigLnc. As for GSE28750, SepSigLnc performed better than all the other models listed, especially logistic regression (AUROC = 0.600) and linear SVM (AUROC = 0.650). Taking the results on GSE57065 and GSE28750 into overall consideration, SepSigLnc has the comparably best performance.

### Validation by children’s data sets

To test the performance of relative expression on various groups of cohorts, we applied SepSigLnc to six children’s data sets GSE8121, GSE9692, GSE13904, GSE26378, GSE4607, and GSE26440. SepSigLnc attained AUROCs over 0.900 on most of the cohorts except GSE9692 and GSE13904(Fig. 3d, supplementary Table S1, and Figure S3 in Additional file). Compared with other machine learning models, SepSigLnc performed better overall. Besides, it surpassed SeptiCyte and was comparative to sNIP. Although the nearest neighbor classifier performed better with AUROC = 0.933 on GSE9692 and AUROC = 0.865 on GSE13904, it almost randomly classified sepsis and normal cases on GSE4607. Neural network achieved AUROC between 0.790 and 0.900 among all the children’s cohorts. Comprehensively, no model can dominate on all cohorts, but SepSigLnc is superior to the others on average.

### Validation by MAS normalization

Validation was also conducted on data with different normalization methods to evaluate the robustness of SepSigLnc. We exerted SepSigLnc and machine learning methods on the same independent adult and children’s cohorts as the previous experiments but normalized by MAS5.0. For the MAS5.0 normalized cohorts, SepSigLnc outperformed machine learning models and other signatures (Fig. 3e, supplementary Table S2, and Figure S4 in Additional file). Furthermore, SepSigLnc appeared to maintain the same level of performance between the two normalization methods, while machine learning methods, SeptiCyte, and sNIP declined on MAS5.0 normalization in comparison to RMA normalization. In consequence, SepSigLnc is superior to the listed machine learning models, SeptiCyte and sNIP across different normalization methods.

### Functional analysis of SepSigLnc

**Fig. 4:**
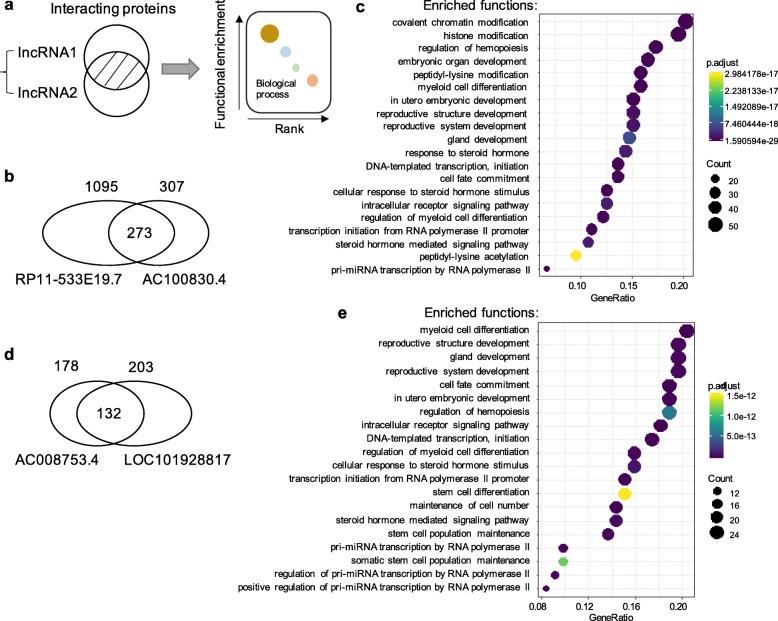
Functional analysis of the lncRNAs in SepSigLnc. **a** The common proteins targeted by a pair of lncRNAs were used to derive the functions of the lncRNA pair. **b** Venn diagram of the proteins targeted by RP11-533E19.7 and AC100830.4. **c** The most significantly enriched biological processes for the overlap proteins targeted by RP11-533E19.7 and AC100830.4. The node color represents the enrichment significance and the node size refers to the number of genes in the functional category. **d** Venn diagram of the proteins targeted by AC008753.4 and LOC101928817. **e** The most significantly enriched biological processes for the overlap genes targeted by AC008753.4 and LOC101928817

We investigated the functional mechanisms of the lncRNAs in SepSigLnc using the rule of guilt by association. First, the interactions between lncRNAs and proteins were established based on the RNAInter database [40]. Then, the common proteins interacted by a pair of lncRNAs were used for function enrichment analysis (Fig. 4a). Specifically, the lncRNA *RP11-533E19.7* interacts with 1095 proteins and *AC100830.4* interacts with 307 proteins, 273 out of which are shared by the lncRNA pair (Fig. 4b). Gene Ontology (GO) enrichment analysis shows the overlapping proteins are implicated in the biological processes of covalent chromatin modification, histone modification, DNA-templated transcription and initiation, cell fate commitment, steroid hormone mediated signaling pathway, etc. (Fig. 4c).

Concentrating on the common target proteins of the *AC008753.4 - LOC101928817* pair (Fig. 4d), we found most of the enriched functions are the same as the *RP11-533E19.7 - AC100830.4* pair, including DNA-templated transcription and initiation, cell fate commitment, steroid hormone mediated signaling pathway, etc. (Fig. 4e). Interestingly, the two lncRNA pairs have an extremely high function similarity, revealing that the SepSigLnc members tend to be implicated in the same pathway associated with sepsis.

**Fig. 5:**
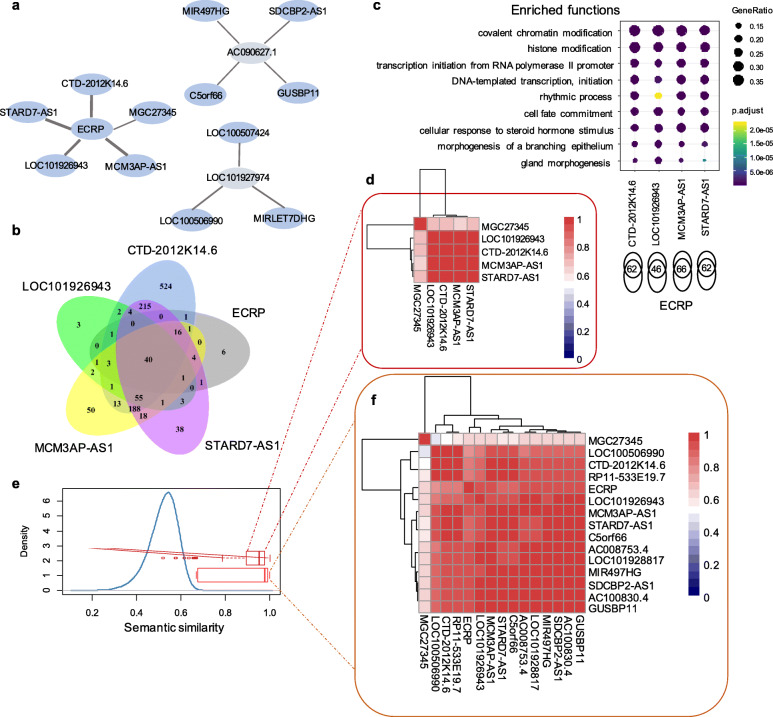
Functional analysis of three connected motifs in SepSigLnc. **a** Twelve out of 14 lncRNA pairs in SepSigLnc compose three connected motifs. **b** Venn diagram of the proteins targeted by ECRP, CTD-2012 K14.6, LOC101926943, MCM3AP-AS1, and STARD7-AS1. **c** The most significantly enriched biological processes for the overlap proteins targeted by ECRP, CTD-2012 K14.6, LOC101926943, MCM3AP-AS1, and STARD7-AS1. **d** The heat map of function similarity between the above five lncRNAs. **e** Semantic similarity (SS) between lncRNAs. Blue line represents the distribution of the SS between all lncRNAs pairs. Bright red boxplot shows the SS between the lncRNAs in SepSigLnc. Dark red boxplot displays the SS between lncRNA ECRP, CTD-2012 K14.6, LOC101926943, MCM3AP-AS1, and STARD7-AS1. **f** The heat map of function similarity between 14 lncRNA pairs in SepSigLnc

On top of the two independent pairs mentioned above, the other 12 pairs composed three connected motifs, including five, four, and three pairs, respectively (Fig. 5a). For instance, the largest motif contains five lncRNA pairs among six lncRNAs, i.e., *ECRP, CTD-2012 K14.6, LOC101926943*, *MCM3AP-AS1*, *MGC27345*, and *STARD7-AS1*. All the five pairs share a single lncRNA *ECRP*. Importantly, a substantially high proportion of target proteins are shared by the five lncRNA pairs (Fig. 5b), indicating that the motif tends to be involved in similar biological processes. Apart from one pair without common target proteins, indeed, the other four lncRNA pairs functions are remarkably similarly based on the enrichment analysis (Fig. 5c). Specifically, *ECRP* separately shares 62, 46, 66, and 62 proteins with *STARD7-AS1*, *LOC101926943*, *MCM3AP-AS1*, and *CTD-2012 K14.6*, the functions enriched by which are pretty consistent, including covalent chromatin modification, histone modification, DNA-templated transcription and initiation, cell fate commitment, cellular response to steroid hormone stimulus, etc. (Fig. 5c).

We further used semantic similarity (SS) to measure the functional similarity between different groups of proteins. As shown in Fig. 5d, the SS scores of all these lncRNA pairs are close to 1 except *MGC27345* (about 0.6), which is significantly higher than the randomly picked up lncRNA pairs (Ranksum test, *P* < 1.34e-22, Fig. 5e). To simulated the SS of random pairs, we grabbed around 20,000 proteins from the RNAinter database [40] and calculated the similarity among any two arbitrarily picked-up protein sets with size ranging from ten to 100 for 10,000 times. Moreover, we evaluated the functional similarity of all the lncRNAs in SepSigLnc and observed a high consistency of them in semantic similarity. Most of the SS scores were over 0.7 (Fig. 5f), which is also significantly higher than the simulated data (Ranksum test, *P* < 1.51e-18, Fig. 5e). Since no target proteins have been detected yet for *AC090627* and *LOC101927974*, the other two motifs were not deciphered in this study. These results indicated that an expression alteration of a pair of lncRNAs may be involved in critical biological pathways that affect sepsis progression through disrupting the balance of the lncRNA-gene regulatory network.

## Discussion

Rapid diagnosis through molecular detection contributes to early intervention for rescue which can reduce the morbidity and mortality of sepsis. Studies revealed that lncRNAs regulate inflammation-related genes and may serve as potential biomarkers or signatures for sepsis diagnosis. Nevertheless, prediction using the absolute expression levels of lncRNAs may deviate since high-throughput platforms are sensitive to various forms of technical bias. The generated continuous measurements were not measurable and comparable, even though they were preprocessed by plausible normalization methods. Using individualized pairwise analysis of gene expression (iPAGE) based on relative expression instead of the absolute abundance, we identified 14 lncRNA pairs named SepSigLnc. The signature’s relative expression of normal controls and sepsis patients are complements to each other respectively and very stable. Importantly, we examined the SepSigLnc on two independent adult as well as six children validation cohorts and demonstrated our model overall outperforms machine learning models and existing biomarkers on the basic of absolute gene expression level. Therefore, SepSigLnc is a reliable and robust signature for the diagnosis and initial evaluation of sepsis. Our results also revealed that the relative expression is more reliable than the absolute abundance in the sepsis high throughput data, which further confirm the previous discoveries [39, 47].

We further tested the diagnosis capability of SepSigLnc across different normalization methods. As shown in Fig. 3e, the performance of SepSigLnc does not reduce (or is even better) when examined on the expression cohorts normalized using another method MAS5.0, whereas it does for other machine learning methods as well as Septicyte and sNIP. To retrieve the absolute abundance of genes from systematic bias of experiments, normalization methods such as RMA and MAS5.0 adjust the expression value according to specific assumptions. Using relative expression of lncRNAs pairs obviates the disturbance brought by normalization, since different normalization methods result in different gene expression patterns, based on disparate assumptions [37, 48, 49].

Theoretically, iPAGE is able to perform cross-platform analysis by integrating expression cohorts from different resources. The absolute expression levels of genes may vary when detected by different high throughput technics such as RNA-seq and microarray, different profiling platforms, or different production batches. iPAGE extracts the relative expression of lncRNA pairs within samples for diagnosis instead of using the absolute abundance and consequently stable across experimental assays and platforms. Validation for the assumption are left for future work.

From the function enrichment analysis of SepSigLnc, the members in a five-lncRNA motif are significantly similar and they share a large amount of interacting proteins, which are implicated in the biological processes of covalent chromatin modification, histone modification, DNA-templated transcription and initiation, cell fate commitment, steroid hormone mediated signaling pathway, etc. (Figs. 4 and 5). In particular, steroid hormone mediated signaling pathway involves in the biological process related to sepsis, which shows high consistence with the previous clinical findings. Sepsis patients often show acute alterations in hormones, including downregulation of hormones such as thyroid stimulating hormone, triiodothyronine, testosterone and estrogen, and upregulation of other hormones such as cortisol and vasopressin [50]. The alteration of hormones partially reflects the physiological response of sepsis patients and it has significant effects on clinical outcomes. For instance, thyroid hormone levels were reduced by different mechanisms during sepsis. Patients with mild illness often have euthyroid sick syndrome, i.e., a decrease in serum levels of triiodothyronine and a mild increase in thyroxine. Moreover, the degree of hormone alteration is associated with disease severity and has been served as a predictor of poor outcome in sepsis [51].

Notably, the function analysis of lncRNA pairs in SepSigLnc implies the regulation mechanism may not only rely on the upregulation or downregulation of a single lncRNA or protein-coding gene, but also through the switch of ordering between lncRNAs in a pair. For instance, *SPI1* and *GATA1* is a well-studied pair in the hematopoietic system. *SPI1* specifies the myeloid lineage, where *SPI1*> > *GATA1*, while *GATA1* specifies the erythroid lineage, featured by *GATA1*> > *SPI1* [52].

Although we constructed a large-scale study covering nine cohorts and 768 samples, all the transcriptome datasets were detected using microarray instead of sequencing techniques. We call for large-scale RNA-seq datasets of sepsis to promote and facilitate the development molecular detection. Additionally, converting the lncRNAs into relative expressions loses information contained in absolute value and hence needs more lncRNA pairs for accurate diagnosis. In the near future, we will validate SepSigLnc through reverse transcription-polymerase chain reaction (RT-PCR) for clinical utility. While the specific role of SepSigLnc in sepsis diagnosis remains unclear, the regulation mechanism needs to be investigated through in vivo experiments such as the cecal ligation and puncture (CLP) model of mouse.

## Supplementary Information

**Additional file 1: Figure S1.** Workflow of machine learning methods. **Figure S2.** The number of reversal lncRNA pairs in SepSigLnc between normal and sepsis samples in eight validation sets. **Figure S3.** AUROC curves on discovery cohort (GSE95233) and eight validation cohorts (others) normalized by RMA. **Figure S4.** AUROC curves on discovery cohort (GSE95233) normalized by RMA and eight validation cohorts (others) normalized by MAS5.0 normalization. **Table S1.** Performance measured by AUROC of SepSigLnc and machine learning methods on independent validation cohorts with RMA normalization. **Table S2.** Performance measured by AUROC of SepSigLnc and machine learning methods on independent validation cohorts with MAS5.0 normalization.

## Data Availability

All the datasets supporting the conclusions of this article are collected from Gene Expression Omnibus (GEO) repository and public available at their stated accession IDs: GSE95233, GSE57065, GSE28750, GSE8121, GSE9692, GSE13904, GSE26378, GSE4607 and GSE26440 (Table 1).

## Abbreviations

ICU: Intensive care unit
PCT: Procalcitonin
lncRNAs: Long non-coding RNAs
SepSigLnc: Sepsis signature lncRNA pairs
AUROC: Area under the receiver operating characteristic
GEO: Gene expression omnibus
RMA: Robust multi-array average
MAS5.0: Microarray suite 5.0
iPAGE: Individualized pair analysis of gene expression
SALPs: Significantly altered lncRNA pairs
SVM: Support vector machine
GPC: Gaussian process classification
GO: Gene ontology
SS: Semantic similarity
RT-PCR: Reverse transcription-polymerase chain reaction
CLP: Cecal ligation and puncture
