# Supplementary material for: Long non-coding RNA pairs to assist in diagnosing sepsis
Source: BMC Genomics. 2021 Apr 16;22:275. doi: 10.1186/s12864-021-07576-4 (PMC8050902; doi:10.1186/s12864-021-07576-4)
Supplement: Supplementary file 1 — Additional file 1: Figure S1. Workflow of machine learning methods. Figure S2. The number of reversal lncRNA pairs in SepSigLnc between normal and sepsis samples in eight validation sets. Figure S3. AUROC curves on discovery cohort (GSE95233) and eight validation cohorts (others) normalized by RMA. Figure S4. AUROC curves on discovery cohort (GSE95233) normalized by RMA and eight validation cohorts (others) normalized by MAS5.0 normalization. Table S1. Performance measured by AUROC of SepSigLnc and machine learning methods on independent validation cohorts with RMA normalization. Table S2. Performance measured by AUROC of SepSigLnc and machine learning methods on independent validation cohorts with MAS5.0 normalization. [file 12864_2021_7576_MOESM1_ESM.docx]

**Long non-coding RNA Pairs to Assist in Diagnosing Sepsis**

Xubin Zheng^1,2^, Kwong-Sak Leung^2^, Man-Hon Wong^2^, and Lixin Cheng^1^*

^1^Shenzhen People's Hospital, First Affiliated Hospital of Southern University of Science and Technology, Shenzhen, China, 518000

^2^Department of Computer Science and Engineering, The Chinese University of Hong Kong, Shatin, New Territories, Hong Kong

* To whom correspondence should be addressed. Lixin Cheng, Email: [easonlcheng@gmail.com](mailto:lixincheng@cuhk.edu.hk,), Phone: +8613392436081

Supplementary Figures and Tables


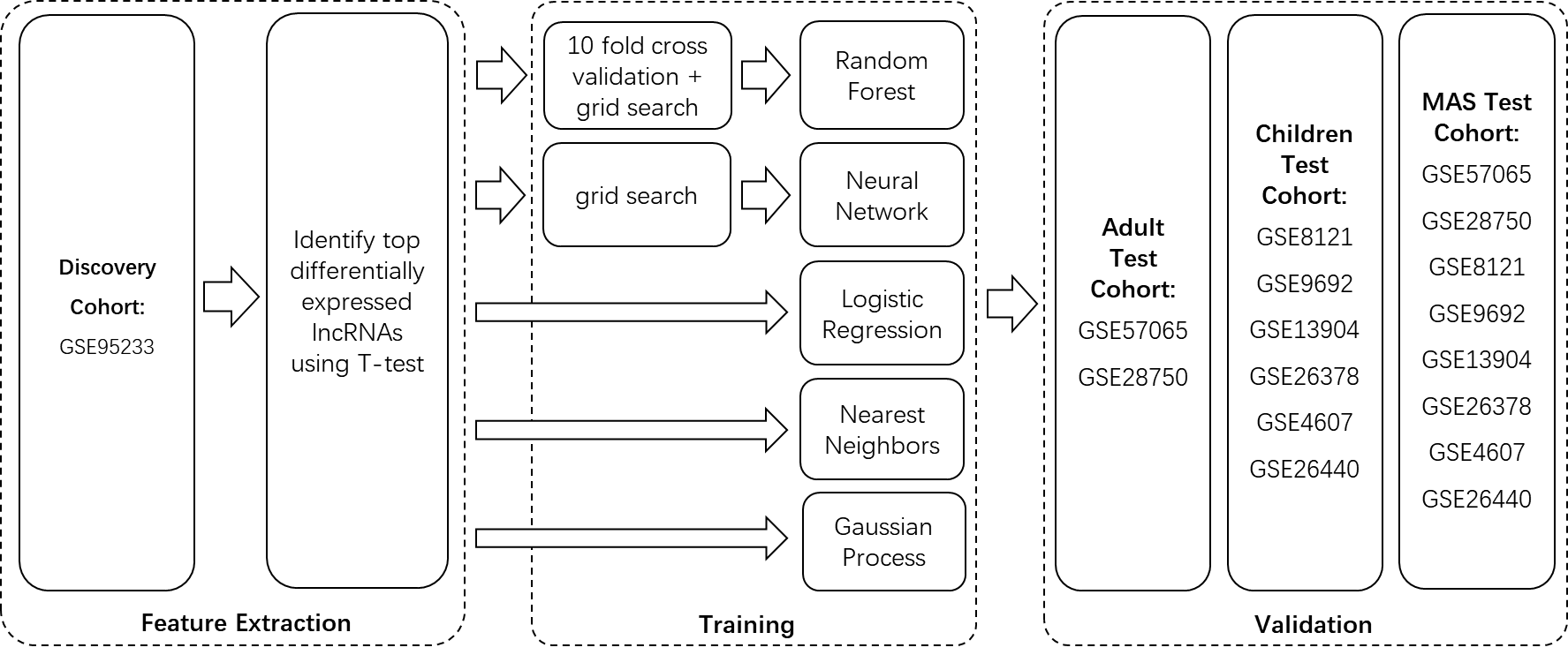


**Figure S1.** Workflow of machine learning methods. In feature extraction, top 19 differential lncRNAs were screened out from discovery cohort GSE95233 using independent T-test. After that, training was conducted on the selected 19 lncRNAs. Ten-fold cross validation and grid search was applied to selecting hyperparameters for random forest. Grid search was used for tuning neural network. Finally, all the machine learning methods were validated on adult and children independent cohorts and MAS normalized cohorts.


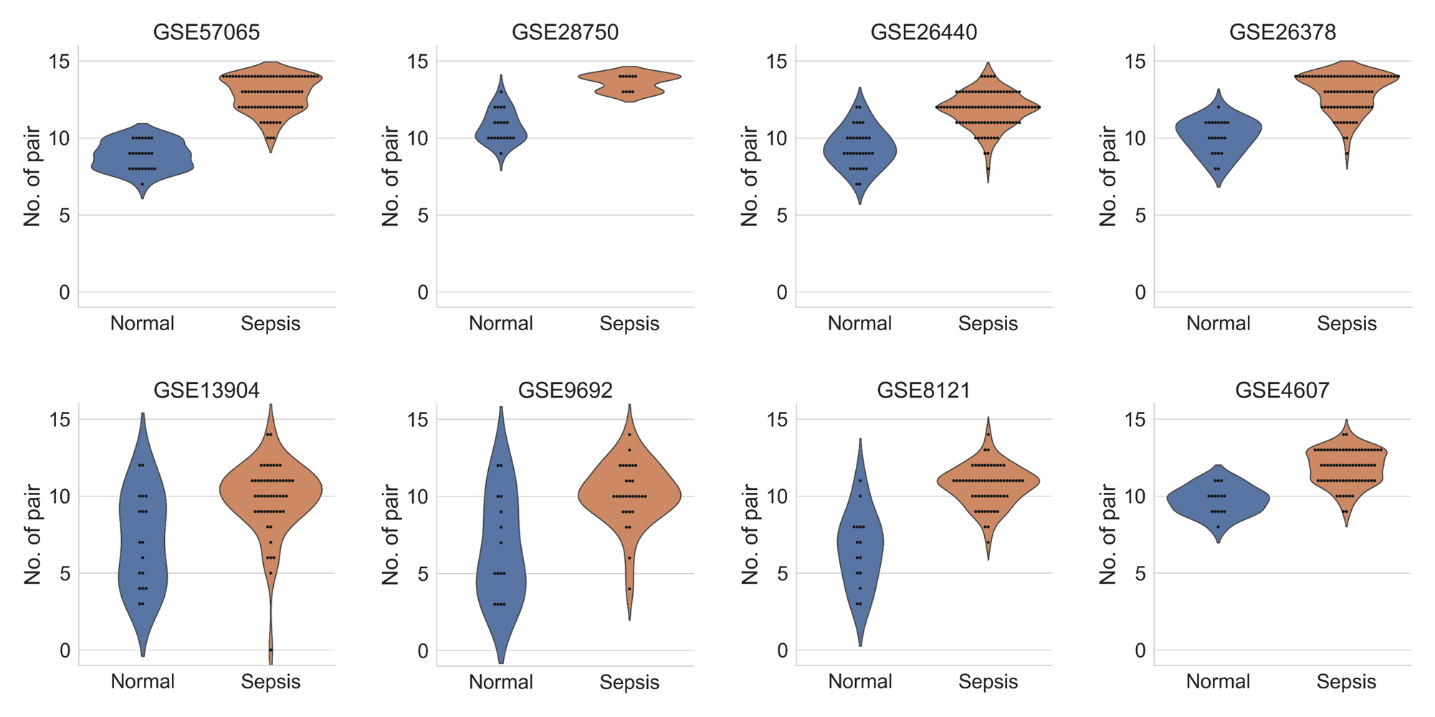


**Figure S2**. The number of reversal lncRNA pairs in SepSigLnc between normal and sepsis samples in eight validation sets. Each point represented the number of reversal lncRNA pairs of each sample in different cohorts.


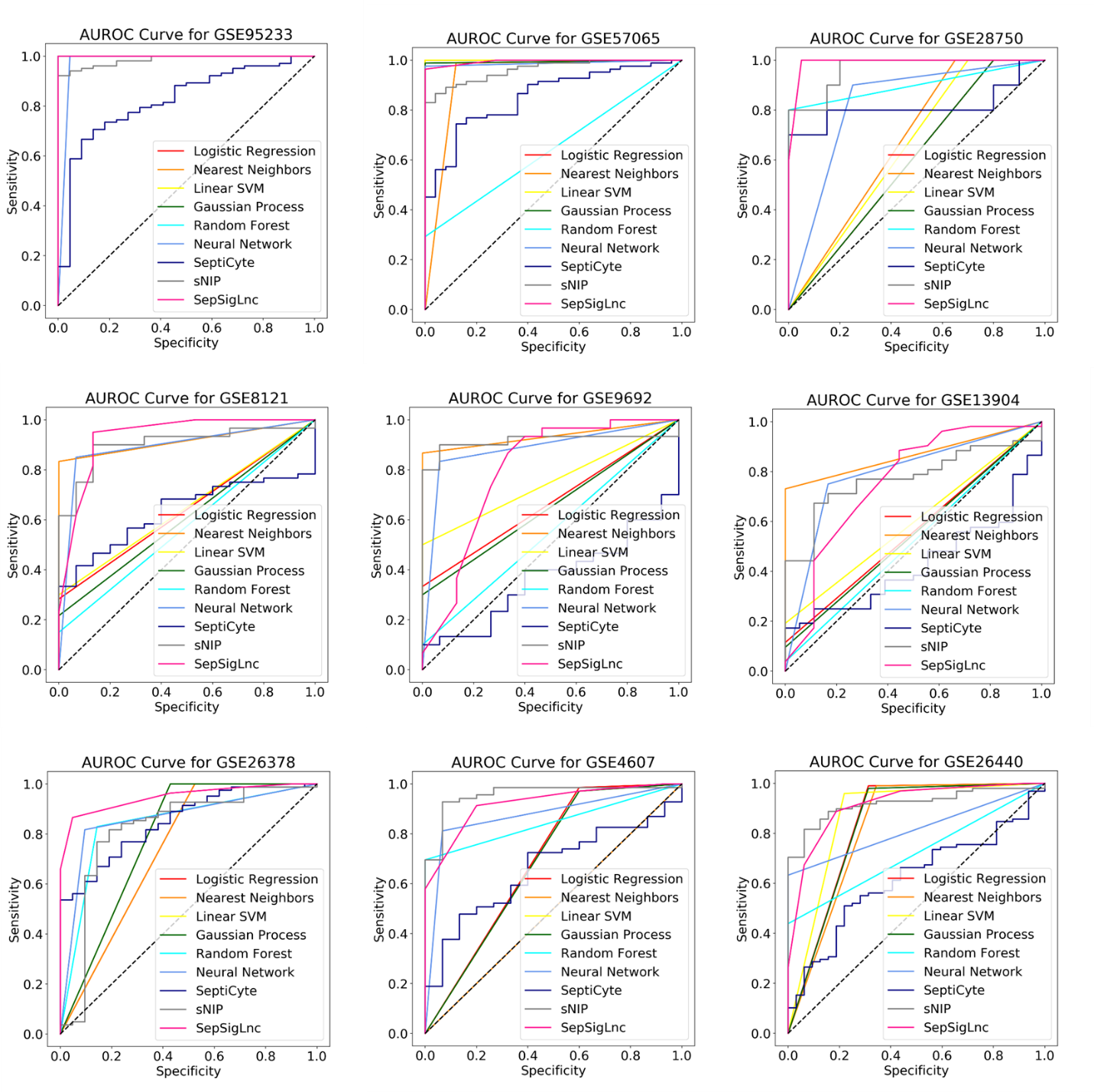


**Figure S3.** AUROC curves on discovery cohort (GSE95233) and eight validation cohorts (others) normalized by RMA.


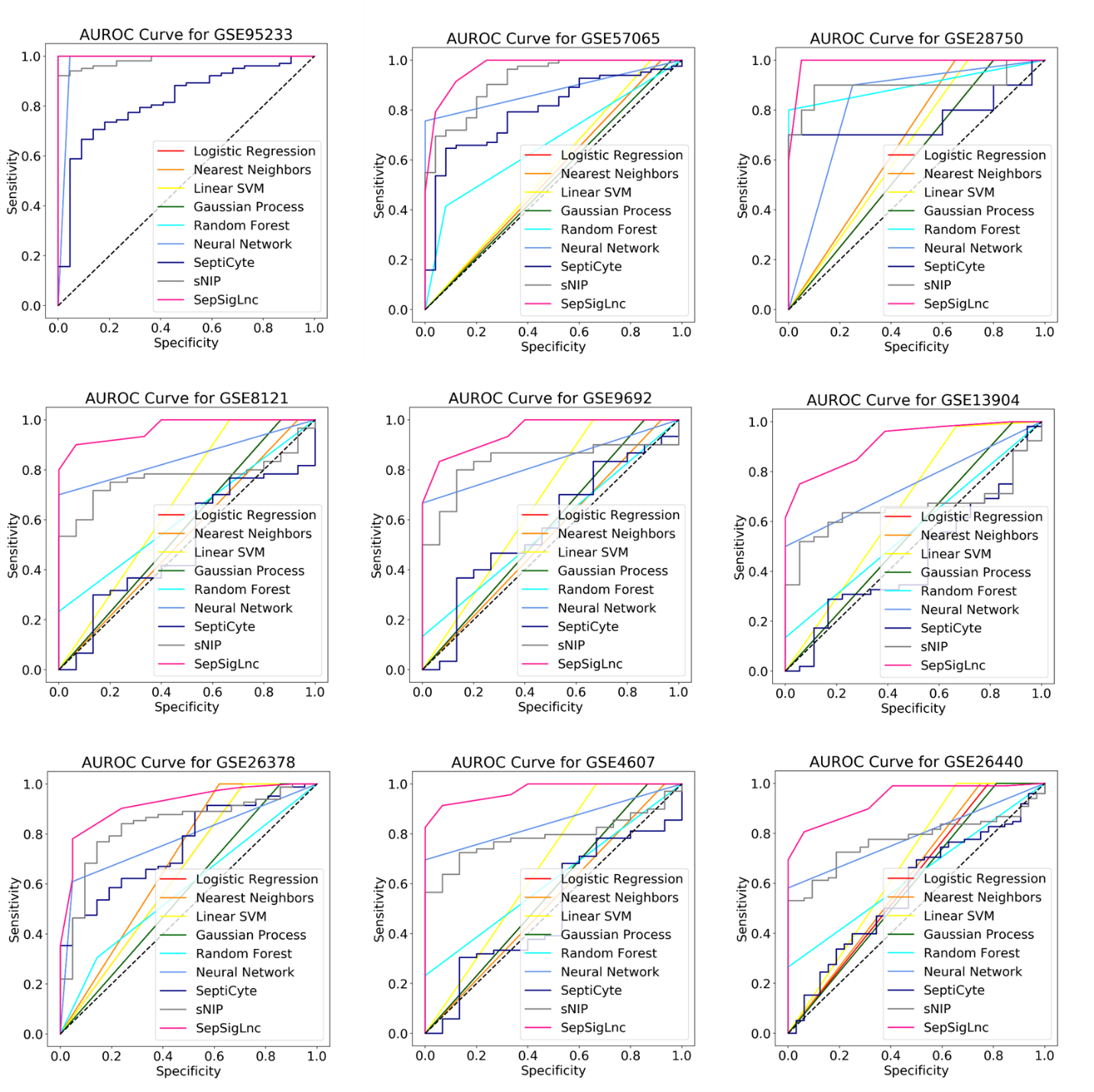


**Figure S4**. AUROC curves on discovery cohort (GSE95233) normalized by RMA and eight validation cohorts (others) normalized by MAS5.0 normalization.

**Table S1.** Performance measured by AUROC of SepSigLnc and machine learning methods on independent validation cohorts with RMA normalization.

|  | **Validation Cohort** | **SepSigLnc** | **Logistic Regression** | **Nearest Neighbors** | **Linear SVM** | **Gaussian Process** | **Random Forest** | **Neural Network** | **SeptiCyte** | **sNIP** |
| --- | --- | --- | --- | --- | --- | --- | --- | --- | --- | --- |
| **Adult** | GSE57065 | 0.995 | **1.000** | 0.934 | **1.000** | 0.994 | 0.646 | 0.988 | 0.854 | 0.962 |
|  | GSE28750 | **0.990** | 0.600 | 0.675 | 0.650 | 0.600 | 0.900 | 0.825 | 0.815 | 0.965 |
| **Child** | GSE8121 | **0.933** | 0.642 | 0.917 | 0.650 | 0.608 | 0.575 | 0.892 | 0.632 | 0.904 |
|  | GSE9692 | 0.794 | 0.667 | **0.933** | 0.750 | 0.650 | 0.550 | 0.883 | 0.367 | 0.916 |
|  | GSE13904 | 0.760 | 0.558 | **0.865** | 0.596 | 0.548 | 0.519 | 0.792 | 0.428 | 0.775 |
|  | GSE26378 | **0.948** | 0.738 | 0.738 | 0.786 | 0.786 | 0.843 | 0.861 | 0.851 | 0.821 |
|  | GSE4607 | 0.921 | 0.693 | 0.500 | 0.686 | 0.686 | 0.848 | 0.872 | 0.624 | **0.957** |
|  | GSE26440 | 0.914 | 0.839 | 0.823 | 0.870 | 0.834 | 0.719 | 0.816 | 0.617 | **0.919** |

**Table S2.** Performance measured by AUROC of SepSigLnc and machine learning methods on independent validation cohorts with MAS5.0 normalization.

|  | **Validation Cohort** | **SepSigLnc** | **Logistic Regression** | **Nearest Neighbors** | **Linear SVM** | **Gaussian Process** | **Random Forest** | **Neural Network** | **SeptiCyte** | **sNIP** |
| --- | --- | --- | --- | --- | --- | --- | --- | --- | --- | --- |
| **Adult** | GSE57065 | **0.969** | 0.540 | 0.540 | 0.560 | 0.520 | 0.667 | 0.878 | 0.797 | 0.920 |
|  | GSE28750 | **0.990** | 0.600 | 0.675 | 0.650 | 0.600 | 0.900 | 0.825 | 0.765 | 0.900 |
| **Child** | GSE8121 | **0.966** | 0.567 | 0.533 | 0.667 | 0.567 | 0.617 | 0.850 | 0.502 | 0.769 |
|  | GSE9692 | **0.950** | 0.567 | 0.533 | 0.667 | 0.567 | 0.567 | 0.833 | 0.571 | 0.831 |
|  | GSE13904 | **0.918** | 0.556 | 0.556 | 0.657 | 0.556 | 0.567 | 0.750 | 0.452 | 0.655 |
|  | GSE26378 | **0.919** | 0.643 | 0.690 | 0.643 | 0.571 | 0.581 | 0.781 | 0.756 | 0.834 |
|  | GSE4607 | **0.972** | 0.567 | 0.533 | 0.667 | 0.567 | 0.616 | 0.848 | 0.503 | 0.788 |
|  | GSE26440 | **0.937** | 0.609 | 0.625 | 0.672 | 0.594 | 0.633 | 0.791 | 0.572 | 0.773 |
